# Pilot Study of the Brief Negative Symptom Scale (BNSS) as a method for evaluation the required form of social service: institutional or non-institutional one

**DOI:** 10.1192/j.eurpsy.2022.2026

**Published:** 2022-09-01

**Authors:** N. Kharitonova, O. Rusakovskaya, O. Papsuev

**Affiliations:** 1 V. Serbsky National Medical Research Centre for Psychiatry and Narcology, Forensic Psychiatry In Civil Process, Moscow, Russian Federation; 2 V. Serbsky National Medical Research Centre for Psychiatry and Narcology, Department Of Clinical, Social And Biological Research On Psychotic Spectrum Disorders, Moscow, Russian Federation

**Keywords:** negative symptom, schizophrénia, BNSS, institutional care

## Abstract

**Introduction:**

Psychiatric care for persons suffering from chronic mental disorders and unable to live independently involves an assessment of their need for a certain form of social service. In Russia patients with schizophrenia account for over 40 % of all persons living in residential facilities for persons with mental disability (Kekelidze, 2020). Their clinical picture is most often determined by negative symptoms, which makes it advisable to use the BNSS scale (Kirkpatrick, 2011).

**Objectives:**

Pilot testing of the BNSS scale in patients, living in residential facilities for persons with mental disability.

**Methods:**

With Russian-language version of the BNSS scale (Mucci, 2019; Papsuev, 2020); CGI-S; Standardized protocol of forensic psychiatric examination in cases of deprivation, restriction, restoration of legal capacity (Kharitonova, 2021) we examined 15 persons (Age: M=54,2; SD=8,6) suffering from schizophrenia and living in residential facilities.

**Results:**

In three subjects the BNSS survey was not possible. The remaining 12 had a total score from 6 to 61 (M = 29.08; Med = 25; Std.Dev. = 17.98) with maximum score in the Asociality subscale (Item 6: M = 3.25; Med = 4; Std.Dev. = 1.76). CGI-S significantly correlated with indicators on the scales «Avolition: inner experience» (r = 0.68, p <0.05), «Blunted affect : vocal expression» (r = 0.64, p <0.05). According to full examination community-based services were recommended for two women with BNSS overall score 6 and 11.

**Conclusions:**

Our pilot study demonstrated that the BNSS can be successfully used as one of methods in comprehensive examination to determine the form of social services.

**Disclosure:**

No significant relationships.

